# Tuberculose hépatique primaire chez un immunocompétent: à propos d’un cas

**DOI:** 10.11604/pamj.2024.48.32.38062

**Published:** 2024-05-30

**Authors:** Yannick Bangda Ekanga, Salma Ouahid, Rachid Laroussi, Chaimae Jioua, Rachida Saouab, Chaymae Faraj, Stephane Ayee, Ibtissam El Ouali, Mohamed Allaoui, Tarik Adioui, Mouna Tamzaourte

**Affiliations:** 1 Service de Gastroentérologie I, Hôpital Militaire, Université Mohammed V de Rabat, Rabat, Maroc,; 2 Service d'Imagerie Médicale, Hôpital Militaire, Université Mohammed V de Rabat, Rabat, Maroc,; 3 Service de Chirurgie Viscérale I, Hôpital Militaire, Université Mohammed V de Rabat, Rabat, Maroc,; 4 Service d'Anatomie et Cytologie Pathologiques, Hôpital Militaire, Université Mohammed V de Rabat, Rabat, Maroc

**Keywords:** Tuberculose hépatique, immunocompétent, cœlioscopie, histologie, cas clinique, Hepatic tuberculosis, immunocompetent, laparoscopy, histology, case report

## Abstract

La tuberculose est une maladie infectieuse courante qui affecte principalement les poumons, peut affecter tout autre organe avec un vaste éventail de manifestations cliniques. Dans l'atteinte hépatique, trois formes peuvent se présenter: tuberculose hépatique diffuse avec tuberculose pulmonaire; tuberculose hépatique diffuse sans atteinte pulmonaire et la tuberculose hépatique nodulaire dite focale/locale qui est une forme très rare et présente un défi diagnostique. Ce cas clinique rapporté chez un jeune marocain, se présentant dans un tableau de colique hépatique évoluant depuis un mois associé à des signes frustres d'imprégnation tuberculeuse. Des lésions nodulaires hépatiques non spécifiques ont été révélées au scanner et à l'imagerie par résonance magnétique (IRM) hépatique. Le diagnostic de tuberculose hépatique focale a été confirmé après analyse anatomopathologique sur biopsies des nodules hépatiques obtenues au décours d'une cœlioscopie. Après début du traitement anti bacillaire bien conduit, une bonne amélioration clinico-biologique a été obtenue.

## Abstract (en)

Tuberculosis (TB) is a common infectious disease that most often affects the lungs, but it can also affect any other organ with a wide range of clinical manifestations. There are three forms of hepatic involvement: diffuse hepatic tuberculosis combined with pulmonary tuberculosis; diffuse hepatic tuberculosis without pulmonary involvement; and nodular or focal/local hepatic tuberculosis, which is a very rare form and presents a diagnostic challenge. We here report the case of a young Moroccan man presenting with biliary colic that had been evolving for a month, associated with a forme fruste of tuberculous impregnation. CT scan and magnetic resonance imaging (MRI) of the liver showed nodular hepatic lesions. The diagnosis of focal hepatic tuberculosis was confirmed based on anatomopathological examination of biopsies obtained during laparoscopy. The patient received antitubercular treatment with good clinical-biological outcome.

## Introduction

La tuberculose maladie infectieuse responsable d'une morbi-mortalité élevée, est un problème de santé mondiale de par son incidence en augmentation continuelle aussi bien dans les pays émergents que dans certains pays industrialisés [1]. Si de nos jours les formes pulmonaires courantes sont assez aisées à diagnostiquer, en revanche les formes extra-pulmonaires notamment abdominales ne le sont pas, car les caractéristiques cliniques et radiologiques manquent de spécificité. Nous rapportons un cas rare de tuberculose hépatique primaire chez un patient immunocompétent, sans autres localisations à distance, en insistant sur la revue de la littérature.

## Patient et observation

**Informations relatives au patient (présentation du patient):** il s'agit d'un jeune homme de 28 ans célibataire sans enfant, militaire en activité. Présente depuis 5 semaines, des douleurs abdominales d'installation progressive plus marquées à l'épigastre et l'hypochondre droite, suivi peu de temps après par une fièvre non chiffrée, des frissons, sueurs profuses dans un contexte d'anorexie et d'amaigrissement chiffré à 6kgs. Notons que le patient n'est pas alcoolo-tabagique, vacciné au BCG ne présentant aucun autre antécédent personnel ou familial contributif.

**Résultats cliniques:** à l'admission, l'examen physique a retrouvé le patient dans un état général altéré, asthénique avec un amaigrissement chiffré à 6kgs sur un mois. Un syndrome inflammatoire à réponse systémique clinique était présent avec comme éléments: une fièvre à 39,1°C, une tachycardie (124 battements/min), une polypnée (22 cycles/min). L'examen pulmonaire et l'exploration des aires ganglionnaires superficielles étaient sans particularité. Au niveau abdominal, une sensibilité modérée à l'hypochondre droite avec une hépatomégalie a été retrouvée.

**Chronologie:** remonte à février 2022 par l'installation d'une douleur abdominale diffuse avec trouble du transite à type de diarrhée-constipation, le tout dans un contexte de conservation de l'état général avec fébricule à prédominance nocturne. Un traitement syntagmatique a été instauré sans succès. L'évolution est marquée par la persistance de la fébricule associée à une anorexie et un amaigrissement progressif chiffré à 12kgs sur trois mois. Devant ce trouble du transit avec fièvre inexpliquée et la dégradation de l'état général le patient sera admis aux urgences pour meilleure investigation.

**Démarche diagnostique:** dès son admission, des bilans ont été réalisés rapportant un syndrome infectieux biologique une hyperleucocytose (17800 élts/mm^3^) à prédominance neutrophile (14000 élts/mm^3^) et une protéine C-réactive élevée à 323 mg/L.

Face à sa douleur abdominale, la lipasémie et la troponine réalisées sont revenues normales respectivement 38 UI/L (VN: <3 78 UI/L) et 4 ng/L (VN: 2 à 16 ng/L). Le bilan hépatique stable avec des ALAT (Alanine amino-transférase) à 22 UI/L (VN: < 40UI/L), des ASAT (Aspartate amino-transférase) à 17 UI/L (VN: < 35UI/L), des GGT (Gamma glutamyl transférase) à 42 UI/L (VN: < 50UI/L), PAL (Phosphatases alcalines) à 115 UI/L (VN: 40- 150 UI/L) et une bilirubinémie normale. La fonction hépatique était normale avec un taux de prothrombine à 78% et une albuminémie à 39 g/L. L'ionogramme sanguin ainsi que la fonction rénale sont revenus normaux. La radiographie du thorax et l'échographie abdominale réalisés sans particularité.

Avec une procalcitonine positive à 4,1ng/L, un bilan infectieux à la recherche du foyer infectieux a été initié entre autres un examen cytobactériologique des urines et des hémocultures lors des pics fébriles à 39°C qui sont tous deux revenus négatifs. Les sérologies hépatites virales B, C et VIH, ainsi que la sérologie de la syphilis réalisée en hospitalisation étaient toutes négatives. La lactate déshydrogénase (LDH) et la béta-2 microglobuline étaient normales respectivement 231 UI/L et 2,28 mg/L. Le GeneXpert à la recherche du *Mycobactérium* sur ces pièces biopsiques était négatif. Le quantiféron était revenu négatif. La recherche du *Mycobactérium* sur les expectorations matinales de 3 jours consécutifs était négative.

**Figure 1:**
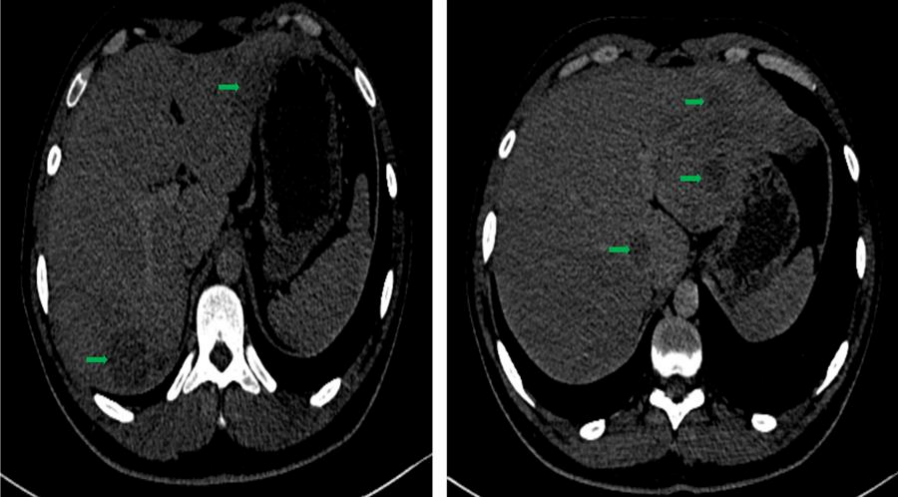
scanner abdominal: foie augmenté de taille (flèche hépatique à 17cm), truffé de multiples lésions hypodenses tissulaire, arrondies bien limitées, non rehaussées après injection du produit de contraste dont les plus volumineuses siégeant aux segments I et VII

Sur le plan morphologique, un scanner thoraco-abdomino-pelvien a montré au niveau de l'étage abdominal, un foie augmenté de taille (flèche hépatique à 17cm), siège de multiples lésions hypodenses tissulaire, arrondies bien limitées, non rehaussées après injection du produit de contraste dont les plus volumineuses siègent au segment I (21 x 16mm) et au segment V (36 x 27mm) (Figure 1). Au niveau des étages thoracique et pelvien, aucune lésion suspecte n'a été mise en évidence. Des premières biopsies hépatiques obtenues par ponction écho-guidée ont révélées à l'examen histologique des lésions hépatiques fibro-inflammatoires subaiguës, sans indice histologique de spécificité ou de malignité.

**Figure 2:**
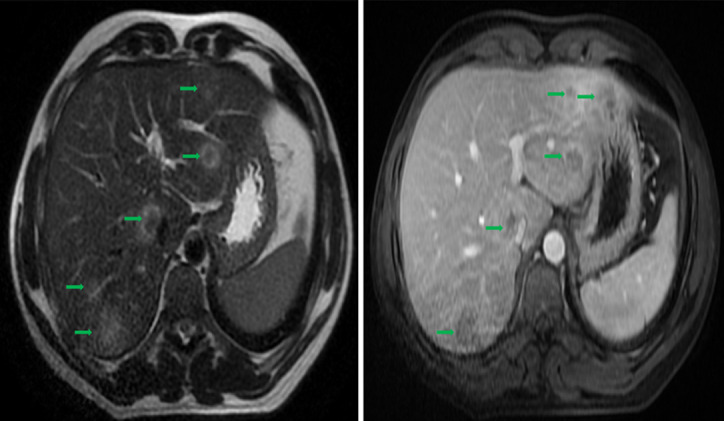
IRM hépatique: foie dysmorphique, truffé de lésions en signal hétérogène T2 entourée d´une paroi en hypersignal T2, rehaussée en périphérie après injection du produit de contraste dont les plus volumineuses siègent aux segments I et VII

**Figure 3:**
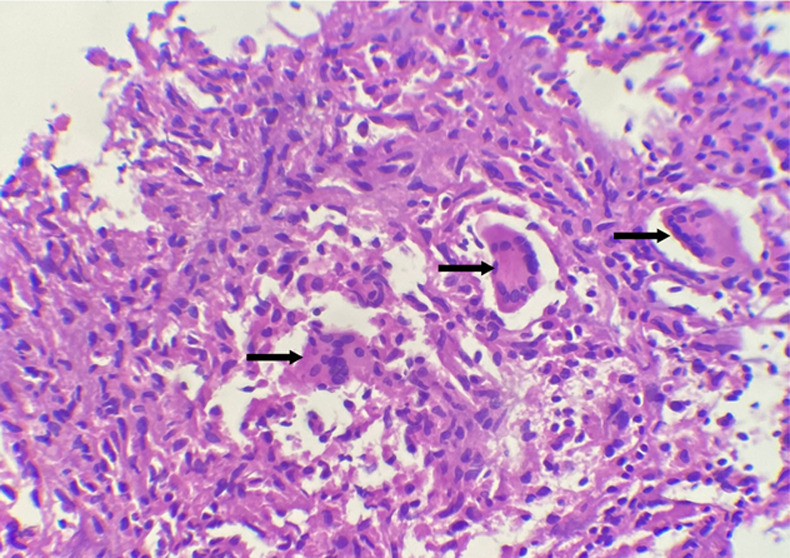
biopsie du nodule hépatique: inflammation granulomateuse épithélio-gigantocellulaire avec début de nécrose caséeuse (HE, Gx400)

Une IRM hépatique à la suite du scanner a objectivé un foie dysmorphique, siège de lésions en signal hétérogène T2 entourée d'une paroi en hypersignal T2, rehaussée en périphérie après injection du produit de contraste dont les plus volumineuses siègent au segment I (20 x 22mm) et au segment V (33 x 31mm) (Figure 2). Aucune adénopathie profonde n'avait été objectivée, aussi bien au scanner qu'à l'IRM. Une cœlioscopie diagnostique réalisée sur les nodules hépatiques, l'examen histologique a retrouvé des granulomes épithélioides et gigantocellulaires de taille variable avec nécrose caséeuse en faveur d'une tuberculose hépatique (Figure 3).

**Intervention thérapeutique:** le patient a été mis sous traitement antituberculeux selon le protocole standard pendant mois à savoir: *Phase 1:* quadrithérapie (Isoniazide + Rifampicine + Pyrazinamide + Ethambutol) en une seule prise orale chaque matin à jeun pendant 2 mois. *Phase 2:* bithérapie (Isoniazide + Rifampicine) en une seule prise orale chaque matin à jeun pendant 4 mois

**Suivi et résultats des interventions thérapeutiques:** dès les premiers jours du traitement, une bonne réponse a été obtenue avec disparition du syndrome inflammatoire à réponse systémique tant sur le plan clinique que biologique. Au bout de trois mois de traitement, un scanner hépatique de contrôle a montré une diminution du nombre et du volume des lésions hépatiques.

**Consentement éclairé:** après un entretien avec le patient lui expliquant le but de notre étude, il a librement donné son consentement éclairé pour permettre aux auteurs d'utiliser toutes ses données utiles à notre article. Ceci a été acté par un document signé par le patient (identité masquée) et le médecin traitant.

## Discussion

La tuberculose digestive constitue l'une des formes les plus communes de tuberculose extra pulmonaire, occupant le 3^e^ rang après la forme pleurale et ganglionnaire. Elle s'observe à tous les âges de la vie, dans les deux sexes avec une sex-ratio homme-femme de 1,1 et se caractérise par un polymorphisme clinique et radiologique. Reposant sur l'imagerie, mais plus spécifiquement sur l'examen histologique et bactériologique des biopsies tissulaires, le diagnostic n'est souvent pas aisé du fait de la complexité qu'elle pose tant sur le plan clinique que morphologique [2]. Dans l'atteinte hépatique, Reed *et al*. en 1990, avaient décrit trois types morphologiques de tuberculose hépatique: tuberculose hépatique diffuse avec tuberculose pulmonaire; tuberculose hépatique diffuse sans atteinte pulmonaire et enfin la tuberculose hépatique nodulaire ou tuberculome hépatique focale ou locale [3].

L'atteinte digestive peut être primitive par ingestion directe de *Mycobacterium* ou secondaire à des lésions pulmonaires très bacillifères par voie hématogène ou lymphatique. En raison de la faible teneur en oxygène dans le foie, défavorable à la croissance des mycobactéries, la localisation focale tuberculeuse pseudo tumorale du foie est rare [4]. La forme pseudo-tumorale du foie serait le plus souvent primitive et retrouvée plus chez l'adulte jeune entre 20 et 40 ans [5]. Chez notre patient de 28 ans, l'atteinte hépatique nodulaire serait due à une confluence de micronodules d'autant plus que l'imagerie montrait d'autres micronodules hépatiques.

Savoir évoquer le diagnostic de tuberculose digestive quel que soit sa localisation est la condition indispensable pour une prise en charge rapide et adaptée, car le pronostic vital est mis en jeu [6]. Le tableau clinique de la tuberculose digestive est en général peu spécifique, se traduisant par un amaigrissement (80%), une fièvre (66%), des douleurs abdominales (100%), une ascite (40 à 100%). On peut également retrouver une constipation (40%), une diarrhée (15%) avec parfois un syndrome dysentérique, voire une forme pseudo-tumorale (5%) [7]. La colique hépatique, la fièvre et l'amaigrissement étaient retrouvés chez notre patient à son admission ainsi qu'une notion de diarrhée aqueuse en cours d'hospitalisation.

La tuberculose des organes pleins (hépatique et/ou splénique), dans sa forme macronodulaire unique ou multiple, peut simuler une lésion tumorale maligne primitive ou secondaire. L'apport de l'IRM dans la localisation abdominale est non spécifique, car cet examen montre également des lésions en hyposignal T1 avec un signal variable T2 au cours des localisations ganglionnaires et viscérales [7].

Devant un aspect hautement suggestif à l'imagerie, un bilan étiologique devrait être entamé pour dépister d'autres localisations pouvant conforter le diagnostic. Si le doute persiste, une ponction guidée sous contrôle échographique ou scanographique avec étude histologique permettrait de poser le diagnostic. En cas de négativité de la ponction écho-guidée ou scano-guidée, la laparoscopie et la laparotomie restent parfois les seuls recours diagnostiques [8]. Seule l'histologie des biopsies profondes des nodules hépatiques issues de la cœlioscopie, nous a permis dans notre cas de poser le diagnostic.

Tout comme dans l'atteinte pulmonaire, le traitement de la tuberculose hépatique repose sur les antibacillaires qui sont la rifampicine, l'isoniazide, l'éthambutol et le pyrazinamide suivant un protocole de quadrithérapie les 2 premiers mois du traitement, puis une thérapie combinée (isoniazide, rifampicine) les 4 mois suivants [9]. Traitement dont notre patient a bénéficié dès le diagnostic posé. Cependant, des difficultés peuvent être rencontrées au cours de ce traitement, d'une part dues à la persistance chronique possible du processus tuberculeux vu l'accessibilité difficile aux mycobactéries intramacrophagiques hépatiques, et d'autre part liées au risque d'hépato toxicité de certains anti bacillaires (isoniazide, rifampicine et pyrazinamide) [9].

## Conclusion

La tuberculose localisée du foie est une affection rare. Toutefois, du fait de l'augmentation de l'incidence de la tuberculose pulmonaire dans les zones endémiques, la possibilité d'une atteint hépatique doit être évoquée chez tout patient présentant une masse hépatique d'allure suspecte à l'imagerie, d'autant plus devant la présence des signes d'imprégnation. Le recours à l'examen histologique des biopsies hépatiques demeure indispensable au diagnostic. Tout comme la forme de tuberculose pulmonaire, la tuberculose hépatique est une maladie potentiellement guérissable. En général, une bonne évolution clinico-biologique est obtenue sous un traitement antibacillaire suivant les protocoles homologués.
